# Delayed diagnosis of pulmonary angiosarcoma in a patient presenting with recurrent hemoptysis and widespread pulmonary nodules: a case report

**DOI:** 10.1186/s13256-026-06211-8

**Published:** 2026-06-14

**Authors:** Hafiz Javed, Arfa Ahmad, Abdul Rehman, Hafiza Noor Ul Ain Baloch, Zohreh Zaki

**Affiliations:** 1 Department of Medicine, TidalHealth Peninsula Regional, Salisbury, MD 21801 USA; 2 Department of Pathology, TidalHealth Peninsula Regional, Salisbury, MD 21801 USA

**Keywords:** Pulmonary angiosarcoma, Hemoptysis, Pulmonary nodules, Heuristics, Clinical reasoning

## Abstract

**Introduction:**

Hemoptysis is a common clinical symptom, which can be caused by a wide spectrum of local, systemic, benign and malignant pathologies. Hemoptysis in conjunction with multiple pulmonary nodules raises suspicion for granulomatous diseases (such as granulomatosis with polyangiitis) or pulmonary metastases from a distant malignancy. Here, we report an unusual case of a patient who presented with subjective fever, hemoptysis and scattered pulmonary nodules, and was subsequently diagnosed with pulmonary angiosarcoma (probable primary pulmonary origin).

**Case presentation:**

A 67-year-old North American White gentleman of Greek ancestry initially presented with fever, cough, and dyspnea, and was found to have reticulonodular pulmonary infiltrates. Over the next 5 months, he developed persistent constitutional symptoms and new hemoptysis. During this interval, he underwent serial outpatient evaluations and was treated empirically for presumed pneumonia and heart failure, followed by unrevealing infectious, interstitial lung disease, and rheumatologic workup. At 6 months, CT demonstrated multiple bilateral pulmonary nodules and masses, and bronchoscopy with transbronchial needle biopsy showed only hemosiderin-laden macrophages, consistent with chronic alveolar hemorrhage. One month later, during a third hospitalization for worsening hemoptysis and hypoxemia, repeat imaging showed progression of innumerable pulmonary nodules and new liver lesions. Ultrasound-guided percutaneous liver biopsy was nondiagnostic, revealing fibrosis, bile ductular proliferation, and granulomatous inflammation without malignancy. During the same hospitalization, thoracoscopic wedge biopsy of the lung was performed. Final surgical pathology, available 1 week later, demonstrated CD34 and factor-VIII-positive malignant cells, establishing the diagnosis of pulmonary angiosarcoma (probable primary pulmonary origin). Thus, definitive diagnosis was made > 7 months after initial presentation. By this time, the patient was profoundly deconditioned and no longer a candidate for systemic chemotherapy; he died approximately 1 month after diagnosis and 8 months after presentation.

**Conclusions:**

Recurrent hemoptysis along with widespread pulmonary nodules is a non-specific presentation, which can be caused by pulmonary angiosarcoma in rare cases. A surgical wedge biopsy is necessary for accurate diagnosis as percutaneous biopsies provide insufficient tissue to delineate the histopathological characteristics pathognomonic of this malignancy. A high index of suspicion and early surgical consultation is necessary to establish an accurate diagnosis and initiate timely treatment.

## Introduction

Hemoptysis is a common clinical symptom reported by 8–15% of all patients presenting to pulmonary clinics, which can be caused by a wide spectrum of pathologies ranging from local and systemic benign diseases to malignant tumors [1]. Mild hemoptysis is most commonly caused by chronic bronchitis or pneumonia, while moderate to severe hemoptysis is usually associated with bronchiectasis, tuberculosis or malignancy [2]. Of all patients with lung cancer, only 20% develop hemoptysis and 0.6–3% have life-threatening hemoptysis [2]. In patients with multiple pulmonary nodules and/or masses along with hemoptysis, differential diagnosis includes pulmonary metastases, septic emboli, granulomatous diseases and atypical infections [3]. Primary pulmonary angiosarcoma is a rare but highly aggressive endothelial malignancy that is seldom suspected early, because its clinical and radiographic features overlap substantially with more common pulmonary disorders. Here, we present an interesting but unfortunate case of a 67-year-old gentleman, who presented with recurrent hemoptysis and multiple pulmonary nodules, and was subsequently diagnosed with pulmonary angiosarcoma of presumed primary pulmonary origin—after a delay of more than 7 months. This case is educationally important, because it demonstrates a prolonged diagnostic course in a patient with recurrent hemoptysis and diffuse pulmonary nodules in whom repeated minimally invasive biopsies were nondiagnostic. Definitive diagnosis required wedge lung biopsy and was established only after a marked clinical decline, precluding chemotherapy. Accordingly, this case adds to the limited literature on pulmonary angiosarcoma by underscoring the importance of maintaining suspicion for this malignancy in patients with persistent hemoptysis, progressive bilateral pulmonary nodules, and chronic alveolar hemorrhage without an alternative unifying diagnosis. It also highlights the importance of avoiding delays in diagnosis as this may preclude candidacy for antineoplastic chemotherapy and the diagnostic uncertainty that may exist when distinguishing primary pulmonary angiosarcoma with intrapulmonary dissemination from angiosarcoma metastatic to the lungs.

### Case presentation

**Fig. 1:**
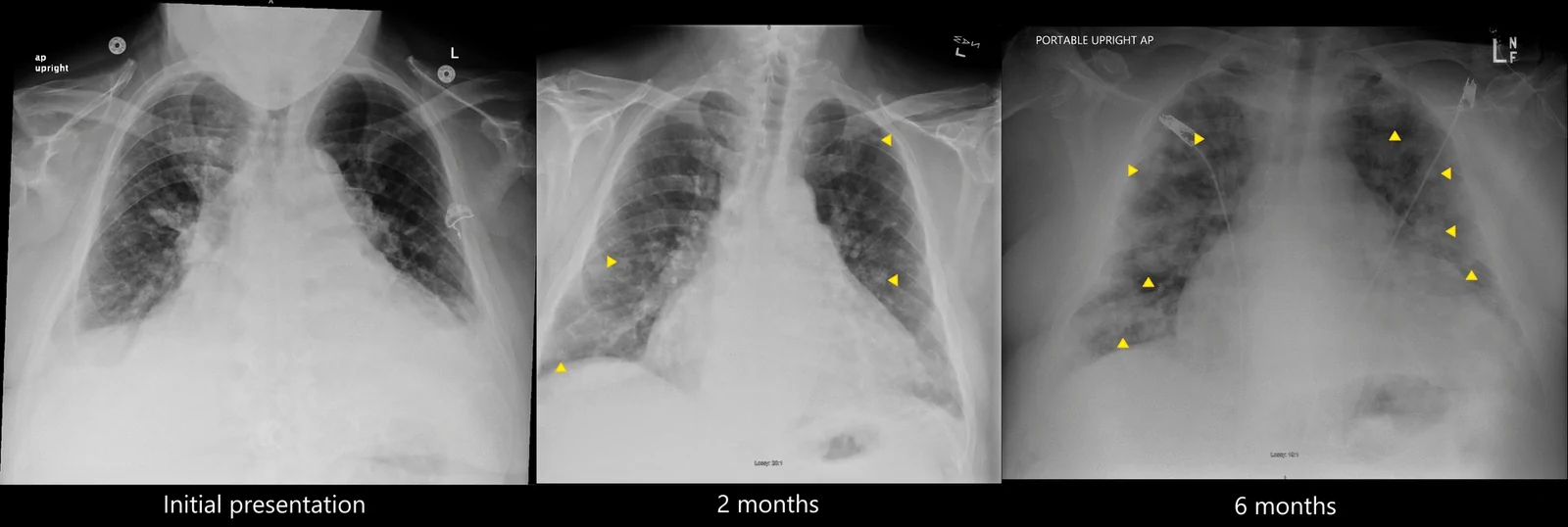
Plain chest radiograph at multiple time intervals demonstrating progression of bilateral scattered pulmonary nodules. No obvious nodules perceptible at presentation (left image), barely perceptible nodules (arrowhead) at 2 months (center image) and bilateral pulmonary nodules (arrowheads) appreciable at 6 months (right image)

**Fig. 2:**
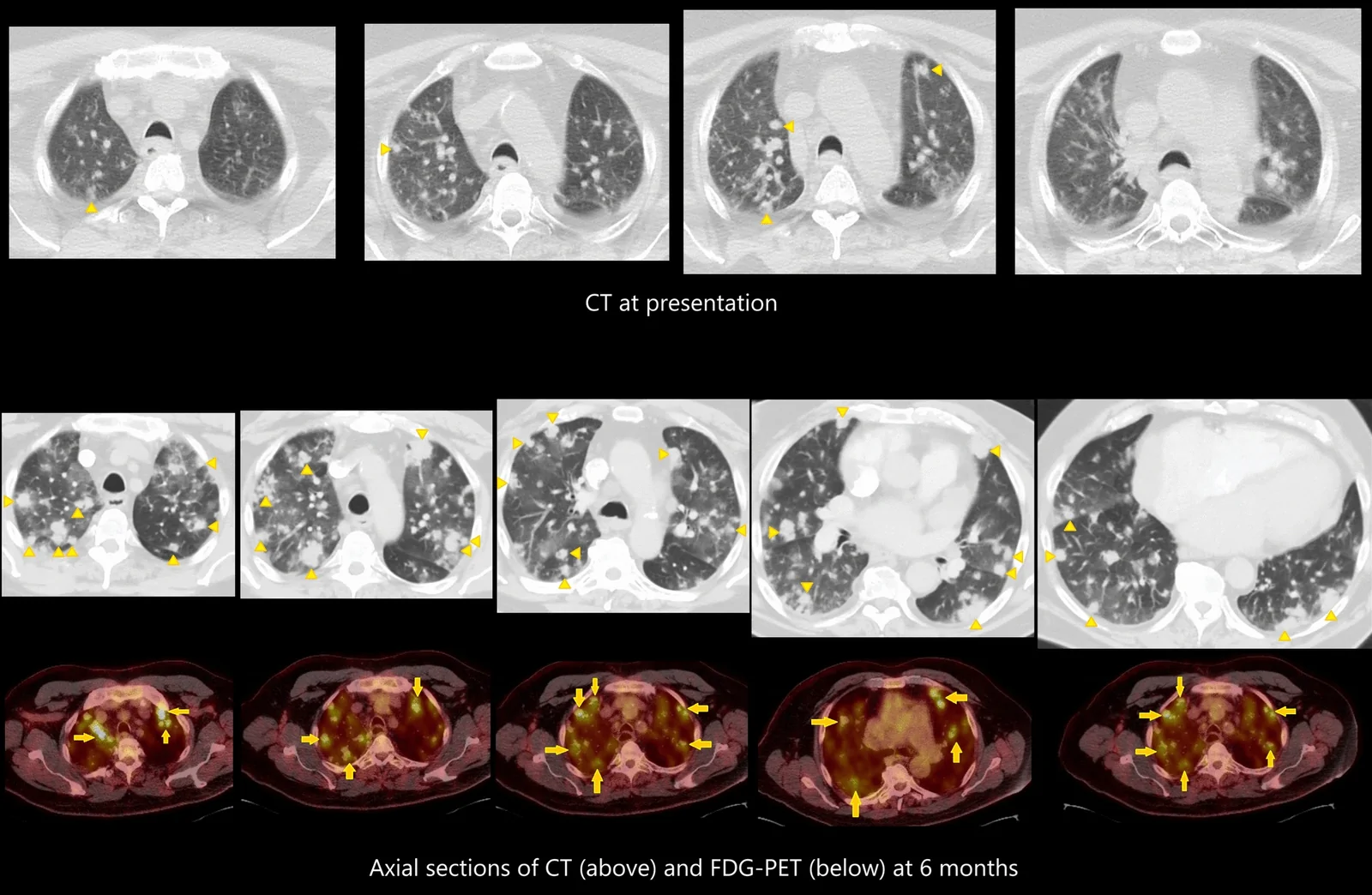
Computed tomography and positron emission tomography of the chest demonstrating bilateral pulmonary nodules. Axial sections of computed tomography at presentation (top row) show only a few scattered pulmonary nodules (arrowheads). At 6 months, axial Computed tomography slices (middle row) show multiple bilateral pulmonary nodules (arrowheads) as well as ground-glass opacities. ^18^fluorodeoxyglucose positron emission tomography fusion images demonstrating some FDG-avid pulmonary nodules (arrows). *CT* = Computed tomography; FDG–PET = ^18^fluorodeoxyglucose positron emission tomography

A 67-year-old North American White gentleman of Greek ancestry initially presented to the emergency department of our institution with complaints of fever, night sweats, leg swelling, shortness of breath and cough for 5 days. He had a known past medical history of paroxysmal atrial fibrillation, chronic diastolic heart failure, obstructive sleep apnea, morbid obesity and testicular cancer as a child. His past surgical history included orchiectomy for testicular cancer and laparoscopic gastric band placement. His family history was notable for a history of colon cancer in his father and a history of breast cancer in his mother. His regular medications included furosemide, rivaroxaban, diltiazem and testosterone undecanoate. He was a lifelong non-smoker and denied any alcohol or illicit drug use. On initial evaluation, he was hypoxic and tachypneic. His physical examination was notable for bilateral pitting pedal edema but negative for any hepatosplenomegaly, lymphadenopathy, skin rash or signs of arthritis. Pulmonary examination did not reveal any inspiratory stridor, fixed wheeze or bronchial breath sounds. Initial chest radiography (Fig. 1) showed reticulonodular pulmonary infiltrates initially interpreted as pulmonary edema. Chest CT (Fig. 2) obtained early during hospitalization demonstrated bilateral basilar infiltrates and scattered ground-glass nodules. He was treated empirically for presumed bacterial pneumonia and decompensated heart failure, and was discharged home after a prolonged hospitalization.

Over the next several months, patient had persistent cough, night sweats, and abnormal chest imaging despite outpatient follow-up and treatment with diuretics and antibiotics. Repeat chest radiography at approximately 2 months remained abnormal (see Fig. 1). At approximately 3 months, he presented again with fever and cough, again without a definitive explanation. By 5 months, he developed mild hemoptysis, reduced appetite, and persistent constitutional symptoms. At this time, his cardiologist stopped his rivaroxaban and his pulmonologist ordered an extensive evaluation for interstitial lung disease. Beta-d-glucan and galactomannan antigen levels were within normal limits. Serologies for *Histoplasma*, *Coccidioides*, *Blastomyces* and *Aspergillus* species were negative. A Quantiferon-TB GOLD test was also negative and serum immunoglobulin levels were within normal limits. Antineutrophil cytoplasmic antibodies (ANCA) including anti-myeloperoxidase and anti-proteinase three antibodies were negative. Antinuclear antibodies resulted positive at a titer of 1:180 along with positive anti-Ro antibody (168 AU/ml). The patient was evaluated by a rheumatologist, who did not find any evidence of a systemic autoimmune disease.

At approximately 6 months after initial presentation, worsening hemoptysis prompted rehospitalization. Contrast-enhanced CT of the chest (Fig. 2) showed multiple scattered bilateral pulmonary nodules and masses, raising concern for malignancy. FDG–PET/CT (Fig. 2) demonstrated multiple FDG-avid pulmonary lesions without a clear extrapulmonary primary site. Robotic bronchoscopy with transbronchial needle biopsy of three pulmonary nodules was performed; cytology and histopathology showed benign lung parenchyma with abundant hemosiderin-laden macrophages, consistent with chronic alveolar hemorrhage, but no evidence of malignancy.

**Fig. 3:**
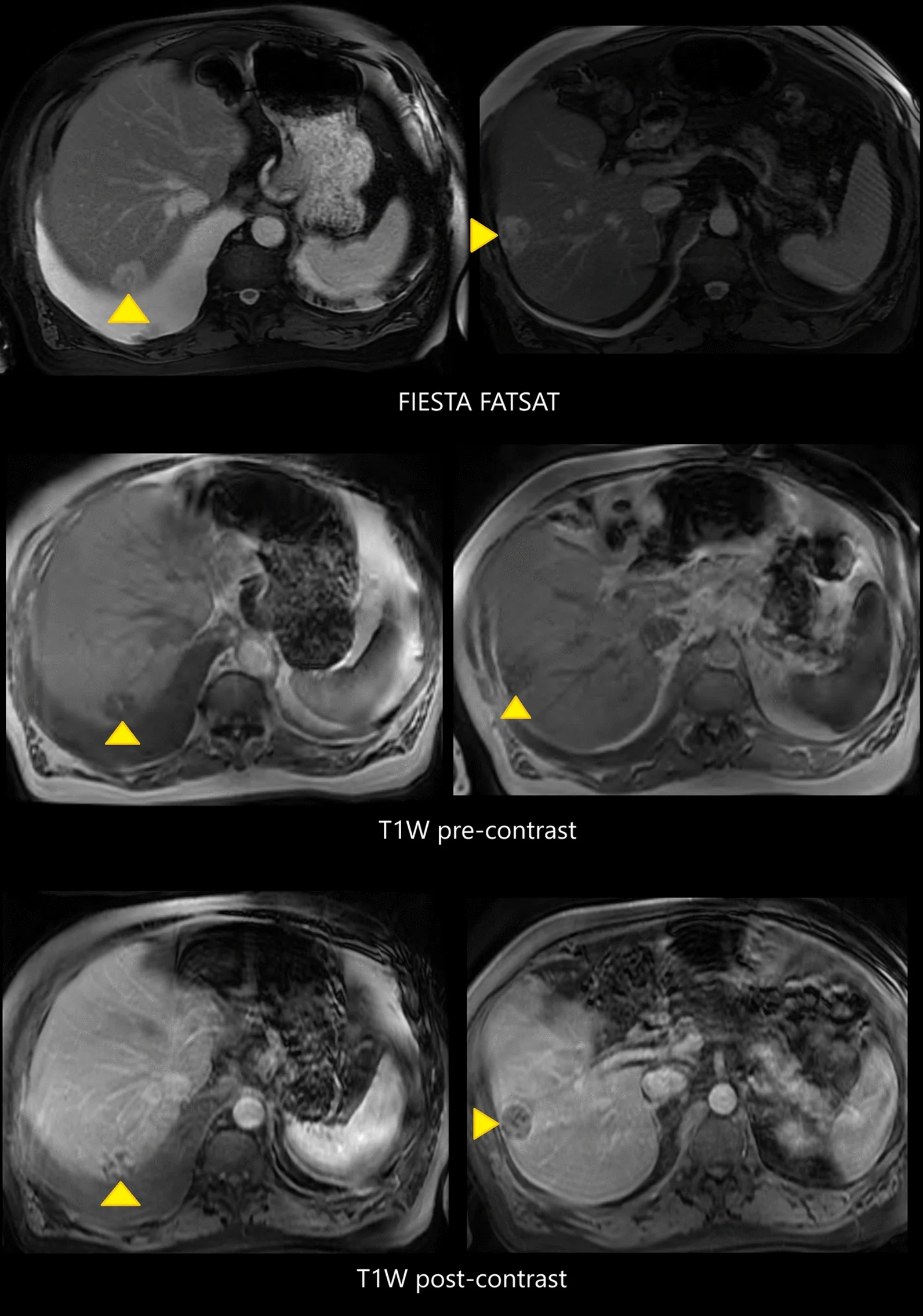
Magnetic resonance imaging of the liver demonstrating liver masses (arrowheads). Axial slices of magnetic resonance imaging showing two liver masses (arrowheads), which are hypointense on T1-weighted imaging and hyperintense on Fat saturation sequences. *FATSAT* = Fat saturation; *FIESTA* = fast imaging employing steady-state acquisition; *T1W* = T1-weighted imaging

Because hemoptysis persisted and respiratory status worsened, he was admitted again at approximately 7 months. Repeat CT chest showed interval increase in the size and number of innumerable pulmonary nodules and identified a new hepatic lesion. Liver MRI (Fig. 3) demonstrated multiple hepatic masses concerning for metastases. Ultrasound-guided percutaneous liver biopsy, however, was nondiagnostic, showing fibrosis, bile ductular proliferation, and granulomatous inflammation without evidence of malignancy.

During the same hospitalization, the patient developed worsening hypoxemia and a large right pleural effusion with lung collapse, requiring chest tube placement and ventilatory support. Pleural fluid cytology and bronchoalveolar lavage remained negative for malignant cells. Despite nondiagnostic minimally invasive sampling, the progressive radiographic pattern of enlarging nodules and masses and persistent hemoptysis strongly suggested an underlying malignant process. Cardiothoracic surgery was, therefore, consulted, and thoracoscopic wedge biopsy of the left lung was performed. Intraoperative frozen section was interpreted as necrotizing granulomatous inflammation.

**Fig. 4:**
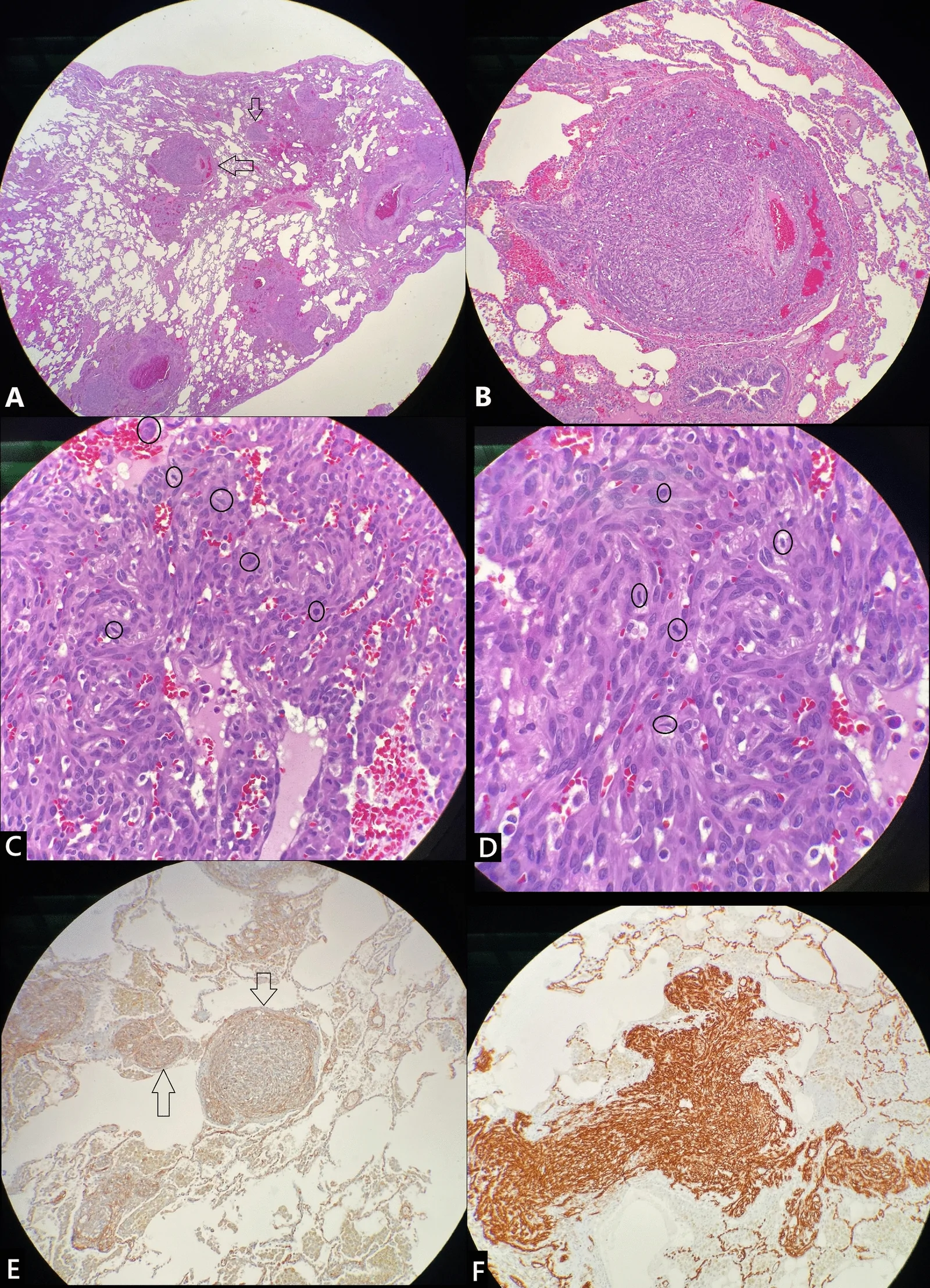
Slides of thoracoscopic wedge lung biopsy for the patient. **A–D** Hematoxylin and eosin stain demonstrating nodular foci of proliferation of malignant spindle cells (hollow arrows) with abundant mitotic figures (circles). Tumor cells stain strongly positive for factor-VIII-related antigen (**E**) and CD34 (**F**) confirming endothelial origin, which is consistent with a diagnosis of angiosarcoma. Tumor cells were negative for cytokeratin AE1/3, actin, desmin, S100, HMB45 and CAM5.2

Final surgical pathology became available 1 week after wedge biopsy, approximately 230 days after initial presentation. The specimen demonstrated multiple nodular proliferations of malignant spindle cells forming irregular vascular channels (see Fig. 4). Tumor cells stained positive for CD34 and factor VIII (see Fig. 4), supporting the diagnosis of primary pulmonary angiosarcoma. Given the presence of diffuse bilateral pulmonary disease and hepatic lesions suspicious for metastases, the malignancy was considered metastatic (stage IV) at diagnosis. The presence of granulomatous inflammation on liver biopsy as well as intraoperative frozen section was likely secondary to a sarcoid-like reaction in the vicinity of cancerous deposits. Transthoracic echocardiography was also negative for any right-sided intracardiac masses. Based on the available clinical data, including the absence of an identifiable extrapulmonary primary tumor and negative transthoracic echocardiography for an intracardiac mass, we considered the findings most consistent with pulmonary angiosarcoma of presumed primary pulmonary origin. However, definitive distinction between primary pulmonary angiosarcoma with diffuse intrapulmonary dissemination and metastatic angiosarcoma to the lungs remains difficult. The hepatic lesions were radiographically suspicious for metastases, but tissue confirmation of hepatic involvement was not obtained.

**Fig. 5:**
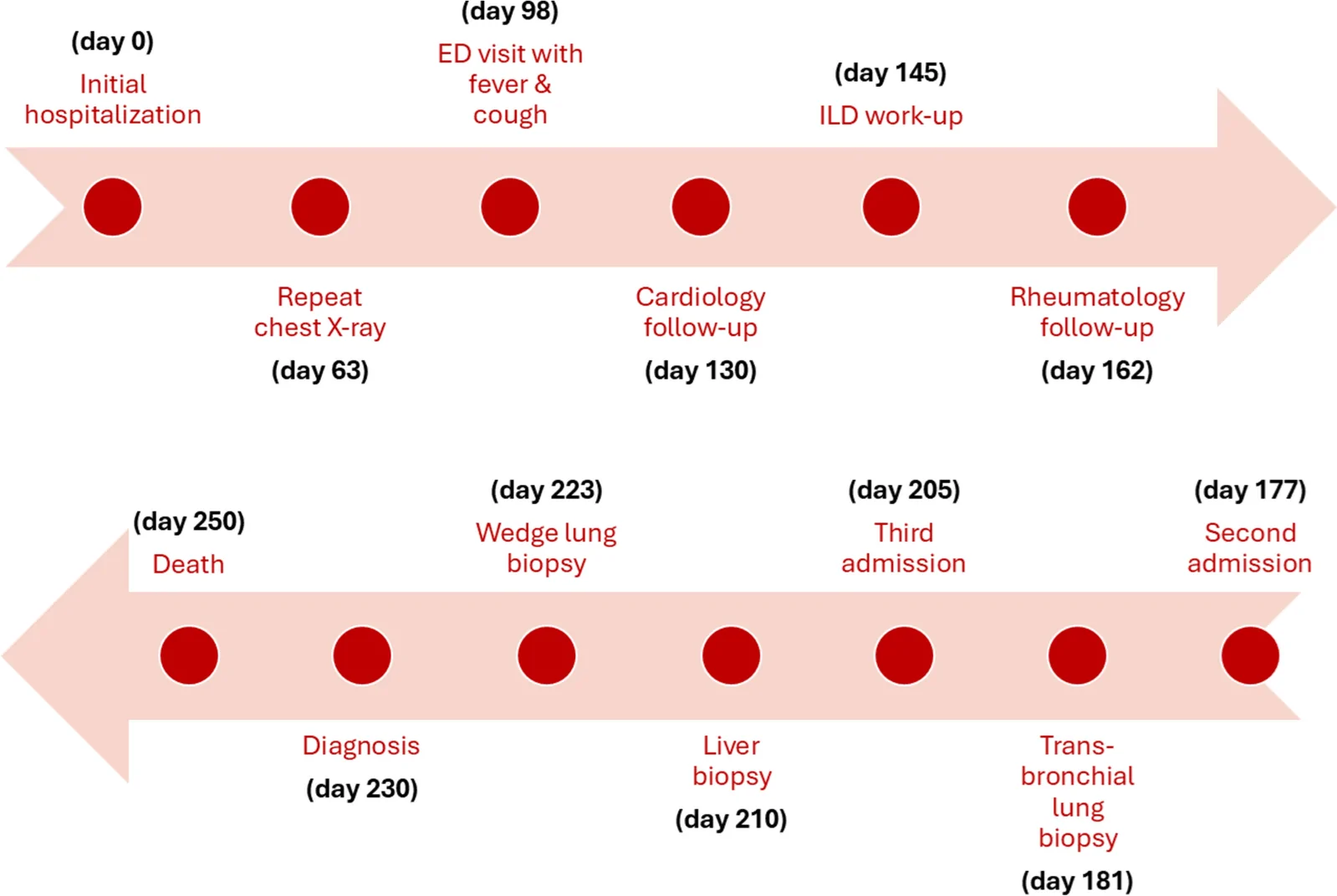
Diagram depicting the timeline of diagnostic workup and clinical course for our patient. After initial hospitalization (day 0), the patient underwent serial outpatient and inpatient evaluations for persistent respiratory symptoms and abnormal pulmonary imaging, including repeat chest radiography (day 63), emergency department reassessment (day 98), cardiology follow-up (day 130), interstitial lung disease evaluation (day 145), and rheumatology follow-up (day 162). Following his second admission (day 177), transbronchial lung biopsy (day 181) was nondiagnostic. During third admission (day 205), liver biopsy (day 210) was also nondiagnostic. Wedge lung biopsy (day 223) subsequently established the diagnosis on day 230, and the patient died on day 250 prior to receiving any antineoplastic therapy

By the time the diagnosis was established, the patient had become profoundly deconditioned as reflected by a Karnofsky Performance Score of 20% and European Cooperative Oncology Group Performance Status of 4, and he was no longer considered a candidate for cytotoxic chemotherapy. He was discharged to rehabilitation, but shortly thereafter returned with massive hemoptysis and worsening respiratory failure. After discussion of goals of care with his family, life-sustaining treatment was withdrawn, and he died approximately 250 days after initial presentation. A complete timeline of major clinical events is summarized in Fig. 5.

## Discussion

The present case documents the enigmatic presentation of pulmonary angiosarcoma of presumed primary pulmonary origin in a 67-year-old gentleman with multiple pulmonary nodules and hemoptysis. The aggressive nature of this rare cancer, coupled with its atypical presentation and late diagnosis, culminated in an unfortunate outcome for the patient at hand. The diagnostic reasoning needed to clinch the correct diagnosis in this case is also highly instructive. In the following lines, we review the clinical features and differential diagnosis of pulmonary angiosarcoma as well as the diagnostic difficulties implicated in diagnosing this entity. This is followed by a discussion of the critical clinical reasoning utilized in this case to arrive at the final diagnosis.

### Clinical features of pulmonary angiosarcoma

Angiosarcoma involving the lungs is rare and highly aggressive with less than 50 cases reported in the English literature [4]. Truly primary pulmonary angiosarcoma is particularly uncommon and can be difficult to distinguish from metastatic angiosarcoma to the lungs. In previously reported cases, patients ranged from as young as 23 years to as old as 85 years [4]. A slight preponderance of male patients was evident in the published cases [4–7]. In our case, the patient was a 67-year-old gentleman. Clinical presentation of pulmonary angiosarcoma is often vague and delayed diagnosis was made in virtually all reported cases [5–9]. The most common clinical symptoms were cough, hemoptysis, shortness of breath and constitutional symptoms [4]. This is consistent with the findings of our case, where the final diagnosis was made after a delay of more than 7 months.

Pulmonary angiosarcomas can arise from the lung parenchyma, pulmonary arteries or bronchi [3]; however, in nearly all reported cases, patients presented with diffuse pulmonary opacities or ground-glass nodules, rather than a single isolated mass [3–9]. Rarely, patients presented with abrupt onset of hemothorax [9] or hemorrhagic pericardial effusion [4] secondary to the aberrant vasculature associated with pulmonary angiosarcoma. In our case, the patient had reticulonodular infiltrates on initial chest radiograph, scattered pulmonary nodules on initial CT chest scan, and subsequently developed progressively enlarging pulmonary masses. Moreover, the patient developed an abrupt right-sided pleural effusion during his third hospitalization, which was likely related to his pulmonary angiosarcoma.

### Differential diagnosis of hemoptysis with pulmonary nodules

Hemoptysis is a common clinical symptom reported by 8–15% of all patients presenting to pulmonary clinics [1]. Mild hemoptysis is most commonly caused by chronic bronchitis or pneumonia, while moderate to severe hemoptysis is usually associated with bronchiectasis, tuberculosis or malignancy [2]. Differential diagnosis for a patient with hemoptysis and multiple pulmonary nodules includes pulmonary malignancies, metastases from distant malignancies, septic emboli and granulomatous diseases, that is granulomatosis with polyangiitis, tuberculosis, sarcoidosis and fungal infections [3]. Presence of a solid nodule surrounded by ground-glass opacities—the so-called “halo sign”—is typically seen in fungal infections, but it can occur in patients with pulmonary angiosarcoma as well [3]. Adjunctive investigations including Quantiferon-TB GOLD, ANCA titers, angiotensin converting enzyme (ACE) level, fungal serologies and *Aspergillus* galactomannan antigen can narrow the differential diagnosis. Bronchoscopy with bronchoalveolar lavage for bacterial, fungal and mycobacterial cultures can be useful for diagnosing infections. However, as demonstrated by this case and previously reported cases [3–7], transbronchial lung biopsies are insufficient to clinch a diagnosis of pulmonary angiosarcoma. Moreover, pulmonary angiosarcoma can co-exist with invasive aspergillosis [4], which complicates the diagnostic process. Unfortunately, there is no reliable way to establish a definitive diagnosis of pulmonary angiosarcoma short of a thoracoscopic or open wedge lung biopsy [3–9].

### Challenges in diagnosing pulmonary angiosarcoma

Pathologic diagnosis of pulmonary angiosarcoma is challenging for several reasons:

- First, percutaneous or transbronchial needle biopsies provide a small amount of tissue, which is insufficient to make an accurate diagnosis of angiosarcoma. In the reported literature, needle biopsies of patients with pulmonary angiosarcoma have led to misdiagnosis of this entity as acute interstitial pneumonia [5], IgG4-related disease [6] and chronic alveolar hemorrhage [7]. In the present case, transbronchial lung biopsy was initially interpreted as diffuse alveolar hemorrhage, although the correct diagnosis was subsequently established by thoracoscopic wedge lung biopsy.
- Second, angiosarcomas can range in appearance from low-grade neoplasms with well-formed anastomosing vessels to high-grade cancers with epithelioid or spindle cells lacking clear vessel formation [3–7]. Moreover, tumor cells may stain positive for cytokeratin, in pleural angiosarcoma [3], or vimentin, in epithelioid variant of angiosarcoma [5]. Wide variation in histopathologic appearance and immunohistochemical markers further complicates the pathologic diagnosis of angiosarcoma.
- Third, angiosarcomas can form slit-like vascular spaces with areas of extensive hemorrhage and blood lakes, which can obscure the primary malignant neoplasm itself [3]. This also explains why low volume, minimally invasive biopsies may preferentially sample hemorrhage, fibrosis or reactive changes.
- Finally, sarcoid-like reactions can occur in the vicinity of tumor cells and result in a misdiagnosis of granulomatous diseases [10]. In our case, percutaneous liver biopsy demonstrated granulomatous inflammation, which was likely a sarcoid-like reaction in response to a metastatic cancer deposit. Moreover, intraoperative frozen section of wedge lung biopsy was also interpreted as necrotizing granulomatous inflammation, which again was sampling of this sarcoid-like reaction.

The histopathologic finding that characterizes pulmonary angiosarcoma is the presence of atypical endothelial cells along vessel walls in conjunction with positive endothelial markers (CD31, CD34 and factor-VIII-related antigen) and negative epithelial markers on immunohistochemistry. In our case, tumor cells were positive for CD34 and factor-VIII-related antigen but negative for cytokeratin AE1/3. In the reported literature, thoracoscopic or open wedge lung biopsy was deemed to be the most reliable method of diagnosis of pulmonary angiosarcoma [3, 4].

### Treatment of pulmonary angiosarcoma

Treatment of pulmonary angiosarcoma is based on extrapolation of treatment regimens for other types of sarcoma. Given the rarity of this neoplasm, evidence-based approaches to treatment are lacking, and a variety of treatment modalities including surgery, chemotherapy and radiation therapy have been employed in previously reported cases [4–9]. Docetaxel–gemcitabine and doxorubicin–ifosfamide were the common chemotherapeutic agents used [4]. In some cases, recombinant IL-2 therapy has been used in conjunction with radiation therapy [4].

### Prognosis of pulmonary angiosarcoma

The prognosis of pulmonary angiosarcoma is universally poor and median life expectancy was less than 6 months in previously reported cases of pulmonary angiosarcoma [4–9]. The dismal prognosis of pulmonary angiosarcoma is a consequence of both the aggressive biology of this cancer as well as the delays that occur in diagnosing this challenging entity. In the present case, our patient was severely deconditioned by the time of diagnosis and could not undergo any cytotoxic chemotherapy. He died within a month after diagnosis and approximately 8 months from his initial presentation.

### What this case adds to the published literature

The principal educational message of this case report is not simply that pulmonary angiosarcoma is rare, but that persistent clinical-radiographic concern for malignancy despite nondiagnostic minimally invasive sampling should prompt reconsideration of the working diagnosis and early pursuit of larger-volume tissue acquisition when feasible. This case adds to the limited literature showing that pulmonary angiosarcoma can be missed on needle- or bronchoscopic biopsy and may initially be interpreted as diffuse alveolar hemorrhage or another nonmalignant process. Because therapeutic options for advanced pulmonary angiosarcoma are limited, early recognition of its initial clinical and radiographic consequences—recurrent hemoptysis, chronic alveolar hemorrhage, progressive bilateral pulmonary nodules, and unexplained constitutional symptoms—may be critical to establishing the diagnosis before rapid clinical decline precludes antineoplastic therapy. Although a single case cannot establish that earlier surgical biopsy would have changed the outcome, it underscores a clinically relevant lesson: delayed definitive diagnosis in rapidly progressive malignancy may narrow or eliminate therapeutic options. For clinicians evaluating unexplained hemoptysis with progressive bilateral pulmonary nodules, pulmonary angiosarcoma should remain in the differential diagnosis, particularly when repeated small-volume biopsies fail to provide a unifying explanation.

### Lessons in critical clinical reasoning

The present case affords three instructive lessons in critical clinical reasoning, which are summarized in the following lines along with an explanation of common cognitive heuristics (Table 1).

**Table 1:** Key lessons in critical clinical reasoning based on this case

Term	Explanation	Relevance to present case
Anchoring bias	Clinician establishes a diagnosis based on initial data and fails to change his initial impressions based on new data that contradicts his initial impressions	Reticulonodular infiltrates attributed to pulmonary edema and heart failure despite worsening radiographic changes after diuretic therapy
Premature closure	A clinician dismisses new clinical data based on presumptions made on the basis of earlier clinical data without considering the full spectrum of differential diagnoses	Fever, cough and dyspnea treated with antibiotics based on a presumptive diagnosis of pneumonia
Occam’s razor	Look for the least number of diagnoses that can explain all observed clinical findings	A unifying diagnosis of primary pulmonary angiosarcoma could explain all clinical, radiological and pathological findings in our case
Hickam’s dictum	Just because a single diagnosis can account for all clinical findings does not preclude the possibility of multiple simultaneous pathologies	Not directly applicable to this case
Red Herring	A piece of clinical data that, despite being “abnormal”, is completely irrelevant to the patient’s actual diagnosis	Positive antinuclear antibody test in the absence of any rheumatologic disease

*Anchoring bias* First, the importance of not anchoring on an initial clinical diagnosis is evident, especially in the face of contradicting clinical data [11]. Anchoring bias is a cognitive error, where a physician establishes a diagnosis based on the first piece of information they receive, without taking into consideration new or contradictory data that may become available later. Anchoring bias often leads to “premature closure”, whereby clinicians may dismiss new or contradictory data due to presumptions made on the basis of initial data. Essentially, physicians end up “anchoring” onto their initial thoughts about a patient and fail to re-consider another diagnosis, even when new data are incompatible with their initial impressions. In the present case, reticulonodular interstitial infiltrates were evident on multiple chest radiographs, which were attributed to pulmonary edema. While this may have been a reasonable interpretation of the initial chest radiograph, persistence of these findings despite being on diuretics should have prompted evaluation for alternative diagnoses. Moreover, the patient had subjective fevers and persistent cough for which he was prescribed multiple courses of antibiotics for a presumptive diagnosis of pneumonia. Again, the lack of response to antibiotics and the persistence of clinical symptoms should have prompted evaluation for alternative diagnoses. In general, anchoring bias can be mitigated by independently assessing a patient’s clinical features without relying entirely on other providers’ impressions and deliberately pausing to consider all potential entites within the differential diagnosis.

*Unifying synthesis* The second important clinical lesson is the correlation and synthesis of a patient’s signs and symptoms, radiographic abnormalities and pathological findings into a unifying diagnosis. Occam’s razor, also known as the law of parsimony, emphasizes that a clinical synthesis should be based on the fewest number of assumptions. This is often contrasted with Hickam’s dictum, which cautions clinicians that a patient may have multiple simultaneous pathologies at the same time. The patient in this case had two biopsies and two cytology reports that were negative for malignancy. Yet, the patient’s symptoms (hemoptysis and constitutional symptoms) and radiographic findings (multiple progressively enlarging pulmonary masses) were strongly suggestive of a malignant process. An accurate clinical diagnosis was only established after a thoracoscopic wedge lung biopsy, which revealed findings of primary pulmonary angiosarcoma. In this case, a single unifying diagnosis was important in formulating a coherent synthesis, which could account for all clinical features.

*Avoiding Red Herrings* The third and last insight from this case is the ability to avoid getting distracted by irrelevant clinical information or falling into rabbit holes. In the present case, the patient had evidence of positive antinuclear antibody and anti-Ro antibody titers; however, this was incidentally found as part of a diagnostic workup for interstitial lung disease. His subsequent clinical course with progressively enlarging pulmonary masses was inconsistent with an autoimmune disease or Sjögren’s syndrome. Thus the positive antinuclear antibody test was a “Red Herring” and falling into this rabbit hole would deviate the clinician from the actual diagnosis. In some ways, this is similar to signal detection theory which emphasizes that a true signal (“relevant clinical data”) needs to be distinguished from mere background noise (“Red Herrings”).

A summary of common cognitive heuristics and the key clinical lessons from this case is provided in Table 1.

## Conclusion

This case highlights an important diagnostic pitfall in primary pulmonary angiosarcoma, a rare and highly aggressive malignancy that may present with recurrent hemoptysis, diffuse bilateral pulmonary nodules, and pathologic evidence of alveolar hemorrhage, thereby mimicking more common inflammatory, infectious, or hemorrhagic lung diseases. This case adds to the limited literature showing that primary pulmonary angiosarcoma can be missed on needle- or bronchoscopic biopsy and may initially be interpreted as diffuse alveolar hemorrhage or another benign pathology. For clinicians evaluating unexplained hemoptysis with progressive bilateral pulmonary nodules, pulmonary angiosarcoma should remain in the differential diagnosis, including cases of presumed primary pulmonary origin when no extrapulmonary primary tumor is identified. Surgical wedge lung biopsy should be considered when repeated small-volume biopsies fail to provide a unifying explanation. Early recognition of these warning features and timely pursuit of surgical lung biopsy may represent the best opportunity to establish a diagnosis, while the patient remains eligible for antineoplastic therapy.

## Data Availability

All the pertinent data pertaining to this case report has been included within the manuscript. For any additional information or queries, please contact the corresponding author, Dr. Abdul Rehman, via email at abdul.rehman_aku@yahoo.com.
